# Delayed-onset verruca vulgaris in tattooed skin

**DOI:** 10.1016/j.jdcr.2026.02.037

**Published:** 2026-02-23

**Authors:** Ananya A. Shah, Christopher J. Yao, Kathleen A. Mannava, Paul J. Blackcloud

**Affiliations:** Department of Dermatology, University of Rochester Medical Center, Rochester, New York

**Keywords:** cutaneous viral infection, human papillomavirus, tattoo reaction, verruca vulgaris

## Introduction

There are many different types of cutaneous eruptions that can result from tattoos, including inflammatory reactions, infections, and neoplastic processes. Viral warts have been reported within tattoos, but most documented cases arise soon after pigment deposition. Although these lesions are often clinically diagnosed, their localization within tattooed skin warrants histopathologic evaluation to exclude other tattoo-related etiologies. The case presented here describes verruca vulgaris arising within a decades-old tattoo, highlighting viral infection as an important consideration in the evaluation of new lesions confined to tattooed skin and emphasizing the role of dermatologists in recognizing tattoo-associated complications.

## Case presentation

A 43-year-old man with a history of opioid use disorder on buprenorphine–naloxone, recurrent deep vein thromboses on apixaban, and resolved hepatitis C presented to his primary care physician with a 2-month history of new spots within a tattoo on his left lower leg that had been placed at age 13 at a tattoo parlor in Miami, Florida. He described progressive “bubbling” and burning of the area, with some pruritus, but no bleeding or pain. He tried over-the-counter topical antibiotic ointment and petroleum jelly at home with no relief. Social history was notable for release from jail 1 year ago, following approximately 30 years of intermittent incarceration. He denied any subsequent reinking, embellishment, or identifiable trauma to the tattooed area.

**Fig 1:**
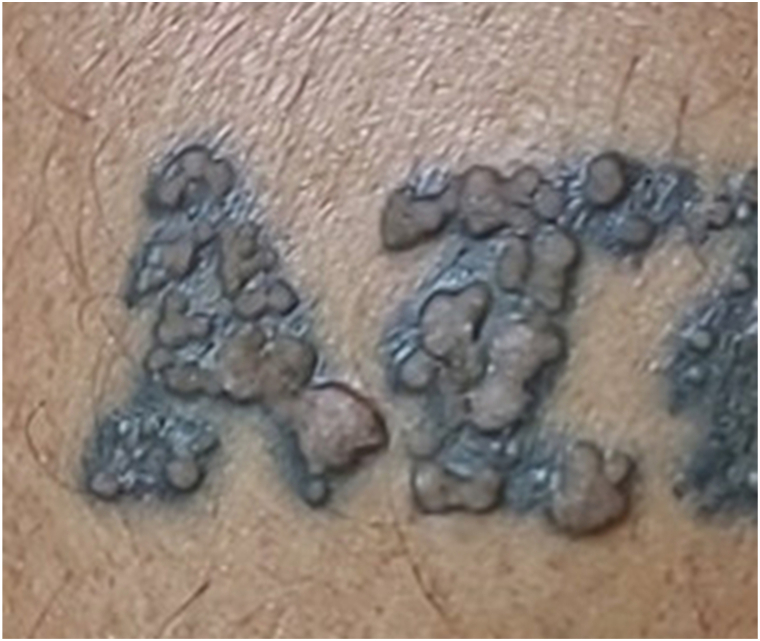
Multiple *brown*, verrucous papules within the *black-blue-gray* pigment of the tattoo on the left lateral lower leg.

**Fig 2:**
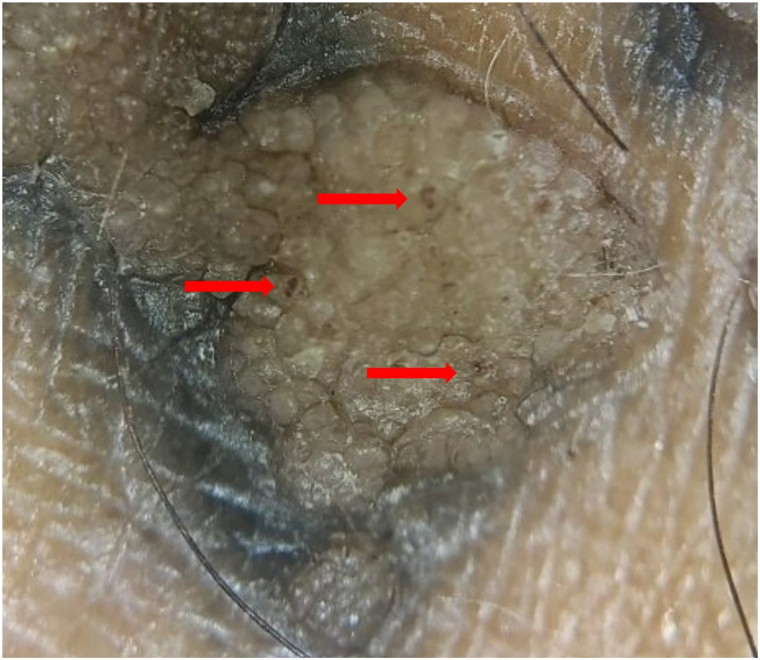
Thrombosed capillaries (*arrows*) in tattoo verruca.

At an initial primary care visit, the lesions were believed to represent an inflammatory or eczematous process. He was prescribed triamcinolone 0.1% and betamethasone dipropionate 0.05% ointments and referred to dermatology. Two months later, the patient presented to dermatology clinic and reported persistent bubbling and pruritus. He denied a decrease in the number of lesions with the application of topical steroids. His other tattoos were unaffected. Physical examination revealed multiple brown, verrucous papules within the black-blue-gray pigment of the tattoo on the left lateral lower leg (Fig 1). Dermoscopy revealed features of verruca vulgaris including multiple pinpoint dotted and thrombosed vessels (Fig 2). Our differential included other potential tattoo-related reactions, including granulomatous, sarcoid, and infectious.

**Fig 3:**
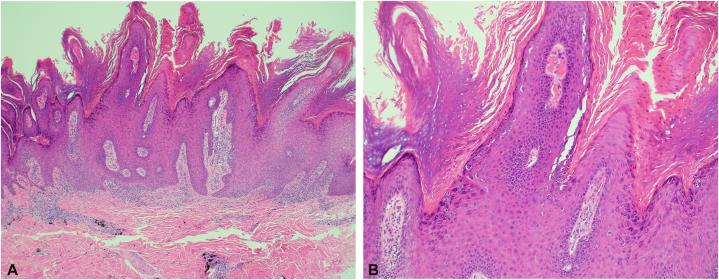
**A,** Verruca vulgaris. Papillomatous epidermis with hypergranulosis, viral cytopathic change, tiers of parakeratosis, and inward bending of the rete ridges. Perivascular exogenous tattoo pigment is visible in the underlying dermis (*left*, H&E, 40×). **B,** Verruca vulgaris. Viral cytopathic change, dilated papillary vessels, and tiers of parakeratosis (*right*, H&E, 100×). *H&E*, Hematoxylin and eosin.

A punch biopsy was performed for hematoxylin and eosin and tissue culture. The biopsy demonstrated papillomatous epidermal hyperplasia and koilocytosis, consistent with verruca vulgaris. Tattoo pigment was present in the dermis, associated with a sparse lymphocytic infiltrate. No granulomatous inflammation was identified (Fig 3, *A* and *B*). Special stains and cultures, including acid-fast bacillus, fungal, and bacterial studies, were negative except for the growth of *Staphylococcus hominis* and *Staphylococcus epidermidis*, consistent with normal skin flora. HIV testing was negative. The patient returned for treatment following the biopsy and received cryotherapy with 2 freeze-thaw cycles to all warts. He reported significant reduction in size and number of warts following this initial treatment.

## Discussion

Viral warts within tattoos are a documented phenomenon with both verruca vulgaris and verruca plana reported. Here, we present a case that represents the longest latency period to date—30 years. A review published by Cohen et al 2021 showed 29 cases of verruca vulgaris and 14 cases of verruca plana within tattoos with latency periods ranging from 1 month to 21 years for verruca vulgaris.^1^ Large-scale series highlight the spectrum of these complications. A multicenter review of 230 tattoo-associated biopsies reported findings ranging from granulomatous inflammation to epidermal hyperplasia and viral changes, noting the diagnostic breadth of tattoo reactions. The most common patterns included granulomatous and lichenoid reactions, but verruca vulgaris was also identified, emphasizing the need to recognize viral infections in this setting.^2^ A comprehensive update on tattoo complications similarly categorized these into inflammatory/hypersensitivity reactions, infectious processes, and neoplastic mimics, noting that black ink sites are disproportionately associated with both granulomatous inflammation and viral infections such as human papilloma virus-induced warts.^3^

There are several proposed mechanisms to explain the prolonged latency period between tattooing and verruca activation. Ruocco et al first described an immunocompromised district, a localized area of altered immune function arising following cutaneous injury, including tattooing.^4^ This is thought to be from persistent disruption of regional lymphatic flow and injury to peripheral sensory nerves, leading to impaired immune trafficking and altered neuro-immune signaling at the affected site. Over time, these changes can compromise effective local immune surveillance.^5^ This is further supported by pathology from the draining lymph nodes of tattoos which show tattoo pigment.^5^ In addition, skin cancers and granulomatous reaction patterns have been described in tattooed skin, further supporting the role of sustained site-specific immune alteration. In all, these studies show that tattooing can act as a nidus for aberrant immune responses, sometimes years after the initial trauma and pigment deposition.^6^

Although verruca arising within tattoos can be diagnosed clinically, histopathologic evaluation should be considered when lesions demonstrate atypical morphology or are confined to the tattoo, since these presentations broaden the differential diagnosis. Biopsy demonstrating papillomatous epidermal hyperplasia with koilocytosis remains diagnostic, as in this case.^2^^,^^7^^,^^8^ Our patient’s verrucous papules localized to a decades-old tattoo highlight the importance of maintaining a broad differential diagnosis for new tattoo-associated lesions. Recognition that tattoo sites can harbor viral, granulomatous, allergic, and even neoplastic changes ensures appropriate use of biopsy and tailored therapy. Management strategies for tattoo-associated verruca vulgaris should balance efficacy with cosmetic outcomes, acknowledging the risk of pigment alteration or scarring.

## Conflicts of interest

None disclosed.
